# The Poor Prognosis of Acquired Secondary Platinum Resistance in Ovarian Cancer Patients

**DOI:** 10.3390/cancers16030641

**Published:** 2024-02-02

**Authors:** Osnat Elyashiv, Natalie Aleohin, Zohar Migdan, Sophia Leytes, Ofri Peled, Ori Tal, Tally Levy

**Affiliations:** 1 Division of Gynecologic Oncology, Department of Obstetrics and Gynecology, Wolfson Medical Center, Holon 58100, Israel; osnate@wmc.gov.il (O.E.); zoharm@wmc.gov.il (Z.M.); ofrip@wmc.gov.il (O.P.); tori@wmc.gov.il (O.T.); 2 Tel Aviv Faculty of Medicine, Tel Aviv University, Tel Aviv 6997801, Israel; nataliea@tlvmc.gov.il; 3 Department of Dermatology, Tel Aviv Sourasky Medical Center, Tel Aviv 6423906, Israel

**Keywords:** recurrent ovarian cancer, platinum resistance

## Abstract

**Simple Summary:**

Patients diagnosed with epithelial ovarian cancer are usually treated with a combination of platinum-based chemotherapy and debulking surgery. Unfortunately, most of them will experience a recurrence of their disease, especially if diagnosed at an advanced stage. Patients with recurrence are typically re-treated with platinum-based chemotherapy if their disease recurs more than 6 months after the completion of previous chemotherapy. These patients are defined as having ‘platinum sensitive’ disease, while patients progressing less than 6 months after previous treatment are defined as ‘platinum resistant’. It is known that patients who do not respond to platinum-based chemotherapy have a poorer prognosis and are expected to have shorter survival. Patients with platinum-sensitive disease will, at some point, become resistant to platinum, with increasing recurrence episodes. This study aims to compare the outcomes of patients who have primary platinum resistance at first treatment versus patients who acquire platinum resistance at a later stage of their illness.

**Abstract:**

Objective: The goal of this study was to evaluate response to treatment and survival in epithelial ovarian cancer patients with acquired secondary platinum resistance (SPR) compared to patients with primary platinum resistance (PPR). Methods: Patients were categorized as PPR (patients with disease recurrence occurring during or <6 months after completing first-line platinum-based chemotherapy) and SPR (patients with previously platinum-sensitive disease that developed platinum resistance on subsequent treatments). Clinico-pathological variables and treatment outcomes were compared. Results: Of the 118 patients included in this study, 60 had PPR and 58 developed SPR. The SPR women had a significantly higher rate of optimal debulking during their upfront and interval operations, significantly lower CA-125 levels during their primary treatment, and a significantly higher complete and partial response rate to primary chemotherapy. Once platinum resistance appeared, no significant difference in survival was observed between the two groups. The median PFS was 2 months in the PPR group and 0.83 months in the SPR group (*p* = 0.085). Also, no significant difference was found in post-platinum-resistant relapse survival, with a median of 17.63 months in the PPR and 20.26 months in the SPR group (*p* = 0.515). Conclusions: Platinum resistance is an important prognostic factor in women with EOC. Patients with SPR acquire the same poor treatment outcome as with PPR. There is a great need for future research efforts to discover novel strategies and biological treatments to reverse resistance and improve survival.

## 1. Introduction

Epithelial ovarian cancer (EOC) is the most lethal gynecological malignancy. Lack of reliable early detection methods and late presentation at diagnosis contribute to the high mortality rate of ovarian cancer [1]. Treatment at presentation includes surgery and platinum-based chemotherapy. When optimal debulking is not possible, neoadjuvant chemotherapy followed by interval debulking is acceptable [2]. Although most patients respond to treatment with platinum and taxane combination chemotherapy, 75–80% of patients who present with advanced disease will experience recurrence within 18 months [3]. The response to platinum retreatment in recurrence appears to be correlated to the time that passed from the completion of the primary platinum treatment [4,5]. Patients progressing during first line platinum-based therapy or within 6 months after completing the treatment are defined as having platinum-resistant disease with response rates to actually any chemotherapy regimen of 10–30% [5,6,7,8,9] and median survival of less than a year [7,8]. Recurrence after >6 months is considered platinum-sensitive, with response rates of 30–90% to re-treatment with platinum-based chemotherapy [6,7,8,10] and a median survival of up to 45 months, depending on the length of time to recurrence. Nevertheless, progression-free survival (PFS) becomes shorter with each recurrence [11], and eventually most patients who were initially platinum-sensitive acquire secondary platinum resistance.

There is a paucity of data regarding the outcome of patients after developing secondary platinum resistance. The goal of this study was to evaluate response to treatment and survival in patients with acquired secondary platinum resistance compared to patients with primary platinum resistance.

## 2. Materials and Methods

The medical records of patients diagnosed with ovarian, primary peritoneal, or tubal cancer who were treated and followed at the Edith Wolfson Medical Center, Israel, between 2000 and 2015, were retrospectively reviewed. The data collected included: date of diagnosis, histology, grade, stage, BRCA mutation status, type of surgery (primary debulking or interval), presence of residual disease at the end of surgery, chemotherapy regimens and number of cycles received, platinum sensitivity, date of recurrences, and patient status at the end of follow-up or death.

Exclusion criteria included tumors of borderline and mucinous histology, patients who refused adjuvant chemotherapy after primary debulking surgery, patients who did not have recurrence during the study period or did not receive subsequent platinum chemotherapy, trial participation, and patients who were lost to follow-up.

Recurrence and response were evaluated according to RECIST 1.1 criteria [12] using computed tomography (CT) or positron emission tomography (PET CT) scans and by the Gynecologic Cancer Intergroup (GCIG) criteria [13]. Patients with primary platinum resistance (PPR) were defined as having recurrence occurring during or less than 6 months after completion of first line platinum-based chemotherapy. Patients with secondary platinum resistance (SPR) were defined as having a primarily platinum-sensitive recurrence occurring ≥6 months after their initial chemotherapy and who then progressed during or less than 6 months after subsequent platinum-based treatment [14].

Progression-free survival (PFS) was defined as the time from the completion of platinum-based chemotherapy to recurrence. Overall survival (OS) was defined as the time from the initial diagnosis to death or the last follow-up. Post-platinum resistance relapse survival (PRRS) was defined as survival from the last dose of platinum chemotherapy to which resistance was acquired to death or the last follow-up.

This study was approved by the Wolfson Medical Center Institutional Review Board (IRB # 0030-17-WOMC, 22 March 2017)

### Statistical Analysis

Descriptive statistics were used to describe patient demographics. Differences between continuous variables were evaluated using the Students’ *t*-test or Mann–Whitney U as needed. Categorial variables were compared using the chi-square test. Kaplan–Meier curves and the log-rank test were used to compare survival. All analyses were performed using SPSS version 25 (IBM SPSS Inc., Chicago, IL, USA).

## 3. Results

**Figure 1:**
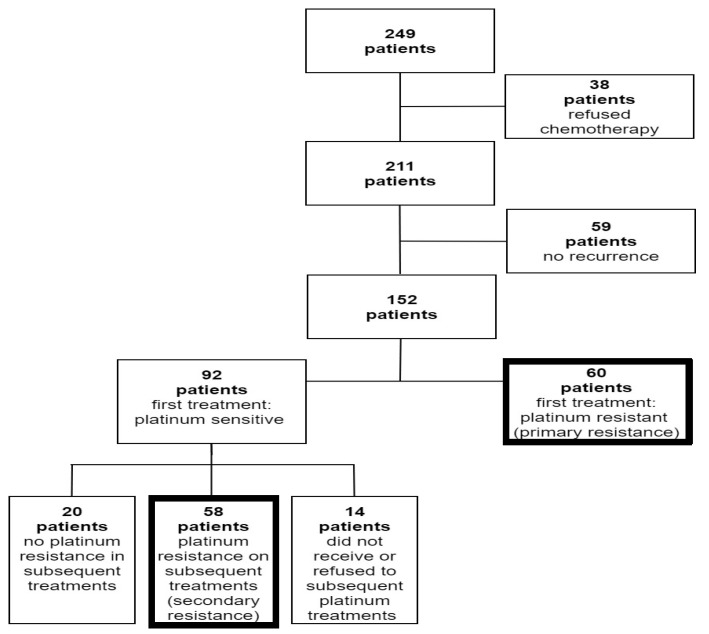
Flow diagram of patient enrollment.

During the study period, 249 patients were diagnosed with epithelial ovarian, tubal, and primary peritoneal cancer (Figure 1). Thirty-eight (15.2%) patients were excluded due to refusal to receive adjuvant chemotherapy, and 59 (23.7%) patients were excluded due to non-recurrent disease. Out of 152 patients who recurred after primary platinum-based chemotherapy, 60 had PPR. Ninety-two patients developed a platinum-sensitive recurrence and were re-treated with platinum chemotherapy. Out of these patients, 58 later developed platinum resistance, which was defined as SPR. An additional twenty women did not develop platinum resistance, and 14 did not receive or refuse subsequent platinum treatments due to side effects or hypersensitivity.

The clinical and pathological characteristics of the PPR and SPR patients are summarized in Table 1. Most patients were diagnosed with ovarian cancer (70.3%), serous type (93.2%), and poorly differentiated disease (74.6%). The majority of patients were diagnosed at stages III (83.1%) and IV (11.8%). CA125 levels were significantly lower in the SPR recurrence group during and at the end of first line chemotherapy.

Information regarding BRCA mutation carriers was available for only 56 patients (47.5%), probably because BRCA testing was not eligible for all patients in the early years of this study. Also, during the study period, no patient received PARP inhibitors.

One hundred and eleven patients underwent either primary debulking surgery (51.7%) or interval debulking (42.3%) after neoadjuvant chemotherapy. The rate of no residual macroscopic disease at the end of surgery was significantly higher in the SPR group compared to the PPR group (62.1% vs. 31.7%, respectively, *p* = 0.014).

**Figure 2:**
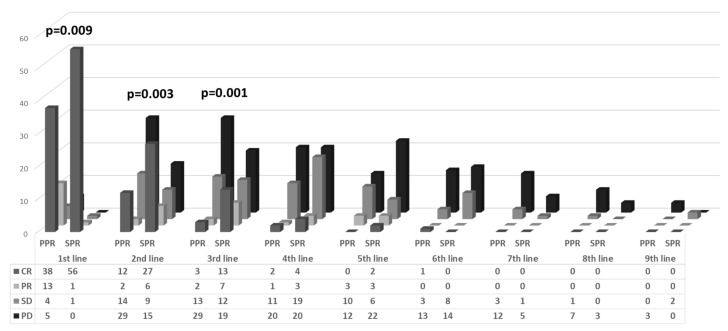
Response to chemotherapy by platinum resistance and treatment line.

All patients were treated primarily with carboplatin and paclitaxel combination therapy. In 82 women (69.5%), 6 treatment cycles were sufficient to achieve complete response (CR): 48 (82.7%) and 34 (56.7%) in the SPR and PPR, respectively (*p* = 0.017). The CR rate to first line chemotherapy was significantly higher in the SPR group (96.5% vs. 63.3%, respectively, *p* = 0.001), while significantly more women in the PPR group showed stable (SD) and progressive disease (PD) compared to the SPR group (14.9% vs. 1.7%, respectively) (Figure 2).

All patients were subsequently treated with chemotherapy for recurrence. In the PPR group, the median number of treatment regimens for recurrence was 3 (range 1–11), mostly topotecan (66.9%), weekly paclitaxel or taxotere (29.3%), pegylated liposomal doxorubicin (PLD, 22.8%), and gemcitabine (14.3%). The SPR patients were treated with carboplatin alone or carboplatin-based chemotherapy (with paclitaxel, PLD, or gemcitabine) for their platinum-sensitive recurrence. Most women in the SPR group (33, 56.9%) received two platinum-based regimens until resistance. In 15 women (25.8%), the platinum resistance developed after 3 platinum-based regimens, in 6 women (10.3%) after 4 regimens, and in 4 more women after 5, 6, 7, and even 9 platinum treatment regimens. After developing platinum resistance, the SPR patients were subsequently treated mainly with PLD (35.9%), topotecan (23.6%), or gemcitabine (10.4%). The SPR patients received a median of 2 chemotherapy regimens (range 0–8) after developing platinum resistance.

In both groups, the CR and partial response (PR) to treatment decreased with each added regimen. In the first, second, and third regimens, there was a significantly higher response rate (CR+PR) in the SPR group compared to the PPR patients, in whom there was evidence of increasing rates of progressive disease (Figure 2). From the fourth chemotherapy regimen onwards, there were no significant differences in the response rates between the SPR and PPR groups.

**Figure 3:**
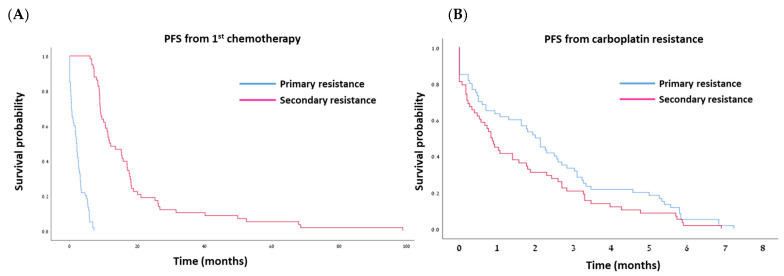
Kaplan–Meier curves for progression-free survival (PFS) after first-line chemotherapy (**A**) and after development of platinum resistance (**B**).

**Figure 4:**
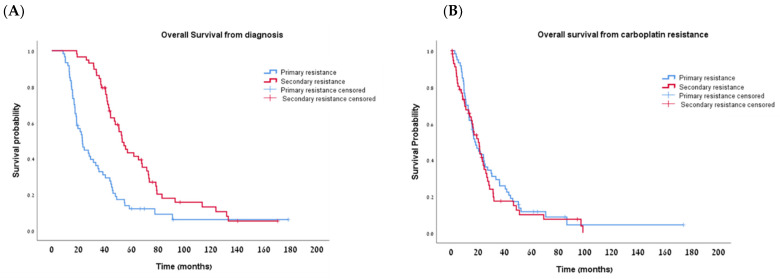
Kaplan–Meier curves for overall survival (OS) from diagnosis (**A**) and from development of platinum resistance (**B**).

Median follow-up was 22.8 months (range: 8.5–178.4 months) for the PPR and 50.5 months (range: 18.8–170.4 months) for the SPR group. As per definition, the median PFS from the last dose of the first carboplatin-based regimen was significantly longer in the SPR group compared to the PPR group: 11.76 vs. 2.0 months, respectively (*p* < 0.001; Table 2; Figure 3A). The median OS from diagnosis was also significantly longer in the SPR group—53.16 vs. 23.03 months in the PPR group (*p* < 0.001; Table 2; Figure 4A). However, once the women developed secondary platinum resistance, the median PFS from the completion of the last platinum-based chemotherapy to the subsequent recurrence was not significantly different between the groups (Table 2, Figure 3B). Similarly, no significant difference was found in the post-platinum-resistant relapse survival between the PPR and SPR groups (Table 2, Figure 4B).

Interestingly, even with platinum resistance, 10 women (16.7%) in the PPR and 6 women (10.3%) in the SPR groups survived more than 4 years after developing platinum resistance.

## 4. Discussion

Platinum-based chemotherapy is still the most active treatment for EOC at the time of diagnosis and recurrence. It is well known that early recurrence after completion of first line platinum treatment is significantly correlated with poor prognosis due to low response rates to any treatment and reduced survival. However, data on response and survival is scarce in EOC patients with platinum-sensitive disease, recurring more than 6 months after initial treatment, and who acquire platinum resistance later in the course of their illness. In our study, 28.4% of ovarian, primary peritoneal, and tubal cancer patients developed PPR, and 27.5% more developed SPR during subsequent treatment. Once EOC patients developed SPR, their outcome was poor and similar to that of patients with PPR. We observed a median PFS of 2 ± 0.24 months in the PPR and 0.83 ± 0.13 months in the SPR groups, and a median PRRS of 17.63 ± 2.6 vs. 20.3 ± 2.7 months, respectively.

Only two previous studies compared treatment outcomes and survival in patients with primary and acquired platinum resistance. Slaughter et al. [15] demonstrated a longer PFS of 21.9 months in patients with SPR compared to 15.1 months for PPR. Although these 7 months of improvement did not reach statistical significance, participation in clinical trials and the number of biological agents received were strong predictors of survival in these platinum-resistant patients. Indeed, more than 50% of their patients participated in clinical trials, thus not necessarily presenting real-world results as in our study. Following the AURELIA trial [16], bevacizumab was approved in combination with weekly paclitaxel at 80 mg/m^2^, topotecan at 4 mg/m^2^, or pegylated liposomal doxorubicin (PLD) at 40 mg/m^2^ as the preferred treatment in platinum-resistant EOC. Trillsch et al. [17] investigated the clinical impact of PPR and SPR on treatment efficacy in patients who participated in this trial. While there was no difference in PFS and PRRS in patients with PPR and SPR in the chemotherapy-only arm, the addition of bevacizumab resulted in a significantly improved outcome in SPR compared to the PPR patients: median PFS of 10.2 and 5.6 months and median PRRS of 22.2 vs. 13.7 months, respectively (*p* < 0.001), pointing to possible different mechanisms in the development of SPR. However, in contrast to our study, the AURELIA study population was limited to patients with ≤2 prior anticancer regimens and excluded tumors that progressed on platinum-based therapy.

At diagnosis, the clinical features were significantly different between our SPR and PPR patients. Significantly higher optimal cytoreduction to no residual disease was achieved (62.1% vs. 31.7, respectively, *p* = 0.014) in women with SPR. The CA-125 levels were significantly lower during and at the completion of first line chemotherapy, and the response rate to subsequent chemotherapy regimens was significantly higher in the SPR vs. PPR groups. Nevertheless, once previous platinum-sensitive patients developed resistant progression, the disease course was similar in terms of response to subsequent chemotherapy, PFS, and PRRS. However, the results of Trillsch et al. [17] and Slaughter et al. [15] suggest that different factors might be involved in the development of PPR and SPR, resulting in a more pronounced response to bevacizumab in women with acquired resistance.

Several mechanisms were suggested to be associated with platinum resistance. Alterations in transporter proteins resulting in reduced uptake, increased efflux, and intracellular inactivation of platinum compounds were all described as resistance-related factors [7,18]. Molecular alterations in DNA repair mechanisms, including mismatch repair (MMR), homologous recombination, and BRCA, can prevent apoptosis by increasing DNA repair mechanisms, resulting in platinum resistance.

A significant proportion of high-grade epithelial ovarian cancers harbor homologous recombination repair deficiencies (HRD), including germline or somatic BRCA mutations. Patients with such mutations have better response rates to platinum-based treatment and longer survival [19,20]. Secondary mutations in BRCA resulting in the restoration of DNA repair function can induce SPR and poor outcomes [21,22]. In the present study, there were significantly more women with germline BRCA mutations (13.8%) in the SPR group compared to only one (1.7%) in the PPR group. Although the numbers are small and we lack information regarding somatic mutations, it is possible that new mutations, leading to one or more of the aforementioned mechanisms, induce SPR in our patient population.

The past decade has introduced the concept of maintenance treatment with PARP inhibitors to patients responding to platinum-based chemotherapy. Primarily evaluated in patients with platinum-sensitive disease after PR and CR to second-line platinum-based chemotherapy, olaparib significantly improved median PFS compared with placebo (8.4 vs. 4.8 months; hazard ratio (HR) 0.35; *p* < 0.001) [23]. A further preplanned interim analysis found that the greatest benefit was observed among BRCA mutation carriers (11.2 vs. 4.3 months; HR 0.18; *p* < 0.0001) [24]. These results were subsequently validated in phase III randomized controlled studies: NOVA [25], SOLO2 [26], and ARIEL3 [27] with niraparib, olaparib, and rucaparib, respectively, leading the regulatory authorities around the world to approve these maintenance therapies for patients with platinum-sensitive, relapsed ovarian cancer, BRCA mutation, or other HRD. Following the promising results in the relapsed setting, several trials showed a significant and meaningful OS benefit when introducing PARP inhibitors at the frontline as maintenance treatment [28,29,30,31,32,33] and currently, PARP inhibitors are approved as frontline maintenance treatment for patients with BRCA and HRD-associated EOC. Since the introduction of PARP inhibitors, there is increasing evidence that these can lead to reconstituted homologous recombination pathways, leading to platinum resistance [34,35]. A retrospective study of BRCA mutation carriers showed that patients progressing on olaparib after a PFS of >12 months had only a 22% response rate to subsequent platinum therapy [36]. Similarly, a retrospective secondary analysis of the SOLO2 patients showed a significantly lower response to subsequent platinum for patients treated with olaparib compared to patients who received placebo [34]. It is important to note that our study population was not exposed to these treatment strategies, and resistance cannot be related to PARP inhibition exposure.

Many novel biological therapies have failed to improve the prognosis of platinum-resistant patients [9]. Immunotherapy was investigated either in combination with chemotherapy or as monotherapy and has failed to show efficacy in this setting. Avelumab, an inhibitor of programmed cell death ligand 1 (PD-L1), was administered to patients with platinum-resistant or platinum-refractory ovarian cancer and did not demonstrate improved median PFS when combined with PLD (3.7 vs. 3.5 months) or median OS (15.7 vs. 13.1 months) compared to PLD alone, or even a poorer median PFS of 1.9 months and a median OS of 11.8 months when given as monotherapy [37]. Nivolumab (a PD-1 inhibitor) also had disappointing results when compared to gemcitabine or PLD as monotherapy, with a median PFS of 2.0 vs. 3.8 months and a median OS of 10.1 vs. 12.1 months [38].

The combination of niraparib and pembrolizumab was evaluated in 62 ovarian cancer patients, most with resistant and refractory disease. Encouraging results were found with an objective response rate of 18% and a disease control rate of 65%, regardless of BRCA mutation or HRD status [39]. Another phase 2 nonrandomized clinical trial assessed the efficacy and safety of a combination of pembrolizumab with bevacizumab and oral metronomic cyclophosphamide in patients with recurrent epithelial ovarian, fallopian tube, or primary peritoneal cancer. Most patients, 75%, had platinum-resistant recurrent disease. Nevertheless, the combination demonstrated clinical benefit in 95.0% and durable treatment responses of >12 months in 25.0% of patients, representing a promising treatment strategy for resistant ovarian cancer recurrence [40]. Recently, Mirvetuximab Soravtansine was shown to significantly improve survival in patients with platinum-resistant, high-grade serous ovarian cancer who had high folate receptor α (FR α) tumor expression (≥75% of cells with ≥2+ staining intensity) as compared to chemotherapy. An objective response rate occurred in 42.3% vs. 15.9% (odds ratio, 3.81; 95% CI, 2.44 to 5.94; *p* < 0.001). The median PFS was 5.62 vs. 3.98 months (*p* < 0.001), and the median OS was also significantly longer (16.46 vs. 12.75 months; hazard ratio for death, 0.67; 95% CI, 0.50 to 0.89; *p* = 0.005), respectively [41]. This successful trial indicates the importance of developing biomarker-targeted therapies. Understanding the molecular shift towards platinum resistance might aid in the development of effective therapies for this poor-prognosis patient population.

In the present study, the response rate (CR+PR) decreased with each additional chemotherapy regimen. Our results indicate that no response was achieved after the fourth recurrence, even in the SPR group. The platinum sensitivity resulted in significantly improved response rates to the first three chemotherapy regimens in the SPR patients, but response to subsequent regimens was equally poor in both groups. Our data are in agreement with previous studies exploring the impact of repeat chemotherapy on recurrent ovarian cancer. It was shown that administration of three lines or more of relapse treatment is not beneficial and has no effect on survival [10,11,42]. Primary platinum sensitivity [10] and platinum sensitivity in subsequent relapses [11,43] were found to have prognostic significance for survival. In our study, once platinum sensitivity was lost, no PRRS advantage was observed.

The strength of this study is that all patients were evaluated, treated, and followed at a single medical center by the same physicians during the study period. The data represent real-world settings with no patients participating in clinical trials using biological agents. That said, the retrospective nature of this study and the long-time span during which different treatment regimens were introduced into clinical practice are clearly its weaknesses.

## 5. Conclusions

The treatment of EOC patients with platinum resistance poses a major challenge. Most clinical trials include platinum-sensitive patients, which are known to have improved outcomes. The few trials involving resistant patients seldom achieve meaningful results. Our data strongly indicate that once platinum resistance develops, the resistance phenotype appears to be the same. However, larger studies evaluating the biologic and molecular properties of the resistance genotype are needed in order to implement new, more effective treatment modalities.

## Figures and Tables

**Table 1:** Clinicopathologic and surgical characteristics of patients with primary and acquired secondary platinum resistance.

Variables	Primary Resistance No. of Patients (%) (N = 60)	Secondary Resistance No. of Patients (%) (N = 58)	Total No. of Patients (%) (N = 118)	*p*-Value
Age (median, years) Range	67.09 28.4–88	66.36 38.6–84.6	66.75 28.4–88	0.123
Type of cancer				0.129
Ovarian	38 (63.3)	45 (77.6)	83 (70.3)	
Tubal	2 (3.3)	3 (5.2)	5 (4.2)	
Primary peritoneal	20 (33.4)	10 (17.2)	30 (25.5)	
Histology				0.584
Serous	55 (91.7)	55 (94.8)	110 (93.2)	
Endometrioid	3 (5%)	2 (3.4)	5 (4.2%)	
Transitional	1 (1.7%)	1 (1.7%)	2 (1.7%)	
Carcinosarcoma	1 (1.7%)	0	1 (0.8%)	
Histological grade				0.059
1	4 (6.6)	1 (1.7)	5 (4.2)	
2	5 (8.4)	0 (0)	5 (4.2)	
3	42 (70.0)	46 (79.3)	88 (74.6)	
Unknown	9 (15.0)	11 (19)	20 (17)	
Type of debulking surgery				0.074
Primary	25 (41.7)	36 (62.1)	61(51.7)	
Interval	29 (48.3)	21 (36.2)	50 (42.3)	
Not operated	6 (10.0)	1 (1.7)	7 (6)	
Debulking				
0 cm	19 (31.7)	36 (62.1)	55 (46.6)	0.014
0–1 cm	20 (33.3)	13 (22.4)	33 (27.9)	
>1 cm	15 (25.0)	8 (13.7)	23 (19.5)	
Not operated	6 (10.0)	1 (1.7)	7 (6)	
Stage				0.038
I	0	2 (3.4)	2 (1.7)	
II	1 (1.7)	3 (5.3)	4 (3.4)	
III	55 (91.7)	43 (74.1)	98 (83.1)	
IV	4 (6.6)	10 (17.2)	14(11.8)	
CA-125 U/mL				
At diagnosis	1767.75	1567.32	1667.53	NS
Middle of treatment *	373.74	146.6	258.1	0.043
End of treatment	190.63	23.74	105.7	0.018
BRCA status				
BRCA+	1 (1.7)	8 (13.8)	9 (7.6)	0.005
BRCA−	20 (33.3)	27 (46.6)	47 (39.8)	
Unknown	39 (65)	23 (39.6)	62 (52.6)	

* after 3 treatment cycles.

**Table 2:** Comparison of survival data in patients with primary and secondary platinum-resistant recurrences.

	Primary Resistance Time in Month (95% CI)	Secondary Resistance Time in Month (95% CI)	*p* Value
Median PFS from first platinum	2.0 ± 0.24 (1.54–2.5)	11.76 ± 2.5 (6.8–16.7)	0.000
Median PFS from resistance	2 ± 0.24 (1.54–2.5)	0.83 ± 0.13 (0.58–1.08)	0.085
Median OS from diagnosis	23.03 ± 2.6 (18.0–28.1)	53.16 ± 2.9 (46.8–59.5)	0.000
Median OS from resistance	17.63 ± 2.6 (12.5–22.7)	20.3 ± 2.7 (14.9–25.6)	0.515

PFS = progression-free survival, OS = overall survival, CI = confidence interval.

## Data Availability

The raw data supporting the conclusions of this article will be made available by the authors on request.
